# Exploring variations in childhood stunting in Nigeria using league table, control chart and spatial analysis

**DOI:** 10.1186/1471-2458-13-361

**Published:** 2013-04-18

**Authors:** Victor T Adekanmbi, Olalekan A Uthman, Oludare M Mudasiru

**Affiliations:** 1 Institute of Public Health, Obafemi Awolowo University, Ile-Ife, Nigeria; 2 Center for Evidence-based Global Health, Ilorin, Nigeria; 3 Warwick - Centre for Applied Health Research and Delivery (WCAHRD), Division of Health Sciences, Warwick Medical School, The University of Warwick, Coventry, , CV4 7AL, UK; 4 Liverpool School of Tropical Medicine, International Health Group, Liverpool, Merseyside, UK

**Keywords:** Stunting, Nigeria, League table, Childhood stunting

## Abstract

**Background:**

Stunting, linear growth retardation is the best measure of child health inequalities as it captures multiple dimensions of children’s health, development and environment where they live. The developmental priorities and socially acceptable health norms and practices in various regions and states within Nigeria remains disaggregated and with this, comes the challenge of being able to ascertain which of the regions and states identifies with either high or low childhood stunting to further investigate the risk factors and make recommendations for action oriented policy decisions.

**Methods:**

We used data from the birth histories included in the 2008 Nigeria Demographic and Health Survey (DHS) to estimate childhood stunting. Stunting was defined as height for age below minus two standard deviations from the median height for age of the standard World Health Organization reference population. We plotted control charts of the proportion of childhood stunting for the 37 states (including federal capital, Abuja) in Nigeria. The Local Indicators of Spatial Association (LISA) were used as a measure of the overall clustering and is assessed by a test of a null hypothesis.

**Results:**

Childhood stunting is high in Nigeria with an average of about 39%. The percentage of children with stunting ranged from 11.5% in Anambra state to as high as 60% in Kebbi State. Ranking of states with respect to childhood stunting is as follows: Anambra and Lagos states had the least numbers with 11.5% and 16.8% respectively while Yobe, Zamfara, Katsina, Plateau and Kebbi had the highest (with more than 50% of their under-fives having stunted growth).

**Conclusions:**

Childhood stunting is high in Nigeria and varied significantly across the states. The northern states have a higher proportion than the southern states. There is an urgent need for studies to explore factors that may be responsible for these special cause variations in childhood stunting in Nigeria.

## Background

Stunting, linear growth retardation is the best measure of child health inequalities as it captures the multiple dimensions of children’s health, development and the environment where they live [1-3]. It is a chronic condition that reflects poor linear growth accumulated during pre and/or postnatal periods because of poor nutrition and/or health. It is more difficult to treat than wasting; which is an acute form of under nutrition. Its relationship to micronutrient deficiencies, obesity (particularly the abdominal type) and chronic diseases makes it an important health hazard even in countries in transition [4]. Depending on the references and criteria used, stunting is defined as somebody with a height-for-age either below the −2 standard deviation from the median or below the 5th percentile in height-for-age. Globally, childhood stunting decreased from 39.7% in 1990 to 26.7% in 2010 and the prevalence is expected to reduce to 21.8% by 2020 [5]. The rate of reduction in childhood stunting is still grossly inadequate to achieve the United Nations’ Millennium Development Goal 1 (MDG1) of eradicating extreme poverty and hunger by 2015 particularly in Africa where stunting has stagnated since 1990 at about 40% and little improvement is anticipated [5]. Available data from the United Nations Children’s Fund estimates that the prevalence of childhood stunting in Nigeria at about 41% [6] which indicates that stunting remains a major public health problem in the country and constitutes a call for action.

Evidently, there exist variations in the patterns of childhood stunting not only across various regions of the world but also within and between local authorities, regional space dimensions and/or countries. Monitoring childhood stunting by measuring the impact of interventions within the locality, region or country remains pivotal for performance evaluation of actions implemented as against set Interventional set goals. This is expected to help shore up the strategic implementation gaps by helping to improve programme design and planning. For ease of assessment and interpretations, data are graphically presented to aid the understanding of policy and decision makers [7]. However, ‘how’, ‘where’, ‘when’ and to ‘whom’ health performance indicators are presented could also determine their level of influence in decision making processes and content. Among the commonly used graphical techniques for presenting performance data are league tables, control charts and spatial analyses [8].

Control charts are meant to differentiate between variation that is expected of a stable process (common-cause variation) and variation that is not expected of a stable process (special-cause variation). Common cause variation is the expected variation attributable to chance. It is part of every process and affects everyone in that process. In contrast, special cause variation is the exceptional variation not attributable to chance, but arising from special circumstances and therefore not affecting everyone in that process.

The developmental priorities and socially acceptable health norms and practices in various regions and states within Nigeria remain disaggregated and with this, comes the challenge of being able to ascertain which of the regions and states identifies with either high or low childhood stunting to further investigate the risk factors and make recommendations for action oriented policy decisions. Considering the challenges of scarce public resources, highlighting the states and regions with greater risk variations in childhood stunting will without doubt help direct appropriately the available resources for maximum impact health interventions. Therefore, the focal point of this study was to examine and describe the variation between states using league table, control chart and spatial clustering of childhood stunting.

## Methods

### Study design

This is a population-based cross-sectional study which used data obtained from 2008 Nigerian Demographic and Health Survey (DHS) [9].

### Sampling technique

The survey used a two-stage cluster sampling technique. The country was stratified into 36 States and the Federal Capital Territory (FCT), Abuja. Administratively, Nigeria is divided into States. Each State is subdivided into local government areas (LGAs), and each LGA is divided into localities. In addition to these administrative units, during the 2006 population census, each locality was subdivided into convenient areas called census enumeration areas (EAs). The primary sampling unit (PSU), referred to as a cluster for the 2008 Nigeria DHS, is defined on the basis of EAs from the 2006 EA census frame [9]. The 2008 Nigeria DHS sample was selected using a stratified two-stage cluster design consisting of 886 clusters [9]. The first stage involved selecting 886 clusters (primary sampling units) with a probability proportional to the size, the size being the number of households in the cluster. The second stage involved the systematic sampling of households from the selected clusters. A representative sample of 36,800 households was selected for the 2008 Nigeria DHS survey, with a minimum target of 950 completed interviews per state [9]. In each state, the number of households was distributed proportionately among its urban and rural areas.

### Data collection

Data were collected by visiting households and conducting face-to-face interviews to obtain information on demographic characteristics, wealth, anthropometry, female genital cutting, HIV knowledge, and sexual behaviour.

### Ethical consideration

This study is based on an analysis of existing survey data with all identifier information removed. The survey was approved by the Ethics Committee of the ICF Macro at Calverton in the USA and by the National Ethics Committee in the Ministry of Health in Nigeria. All study participants gave informed consent before participation and all information was collected confidentially.

### Variables

#### Outcome variable

Nutritional status was measured by height-for-age z-scores (HAZ). A HAZ is the difference between the height of a child and the median height of a child of the same age and sex in a well-nourished reference population divided by the standard deviation in the reference population. Childhood malnutrition (stunting) was defined as height for age below minus two standard deviations from the median height for age of the standard World Health Organization (WHO) reference population [10,11].

### Statistical analyses

#### League table

We compared graphically childhood stunting for each state, plotting the states in rank order. The uncertainty associated with the ranks was then calculated by using the simulation procedure described in the Marshall EC [7]. League tables are used to display comparative rankings of performance indicator scores for several similar groups. They can be used to identify few groups whose indicator scores are higher or lower than expected and to show a range of variations between groups.

#### Funnel plot

We generated scatter plots of performance, as a percentage, against the number of stunted under-fives (the denominator for the percentage). The mean state performance and exact binomial 3 sigma limits were calculated for all possible values for the number of cases and used to create a funnel plot using the method described by Spiegelhalter [12,13].

#### Exploratory spatial data analysis (ESDA)

Local measures of spatial association provide a measure of association for each unit and help identify the type of spatial correlation—these are implemented using the Local Indicators of Spatial Association (LISA) [14,15]. The Local Moran's *I* is used as an indicator of local spatial association. The local measure of Moran’s I is defined as:

$$I_{i}=\frac{n}{\left(n-1\right)S^{2}}\left(x_{i}-\bar{x}\right)\sum_{j}w_{\mathrm{ij}}\left(x_{j}-\bar{x}\right)$$

Where

•*x*_*i*_ is the value of the childhood stunting at a particular state,

•*x*_*j*_ is the value of the childhood stunting at another neighbouring state,

•$\bar{x}$ is the average childhood stunting,

•*w*_*ij*_ a weight that denotes the proximity between states *i* and *j,*

•*n* the number of states, and

•*S*^*2*^ is the variance of the observed values of childhood stunting

The Moran significance map builds on the Moran scatterplot and incorporates information about the significance of “local” spatial patterns. The Moran scatter plot helps identify the nature of spatial autocorrelation between states and can be categorized into:

•Spatial ‘hot spot’ clusters*:* high value of childhood stunting in a state, neighbouring states have high values of childhood stunting.

•Spatial outliers: low value of childhood stunting in a state, neighbouring states have high values of childhood stunting and high value of childhood stunting in a state, neighbouring states have low values of childhood stunting.

•Spatial ‘cold-spot’ clusters: low value of childhood stunting in a state, neighbouring states have low values of childhood stunting.

After computing the appropriate statistics from the smoothed rates, a Monte Carlo Randomization (MCR) procedure was used to recalculate the statistics from the randomized data observations to generate a reference distribution using 999 permutations [14,15]. The p-values were computed by comparing the observed statistics to the distribution generated by the MCR process and significance level was set as .001 [14,15].

### Software

Values for league table and ranking were generated in WinBUGS [16]. Funnel plot was generated in Stata for windows version 12 [17]. Exploratory spatial data analysis (ESDA) was implemented through the GeoDa software [18,19].

## Results

A total of 8,093 out of the 28,647 under-five children included in the 2008 Nigerian DHS who were found to be stunted (about 43.0% male and 38.4% female) were assessed in this study.

### League table

**Figure 1:**
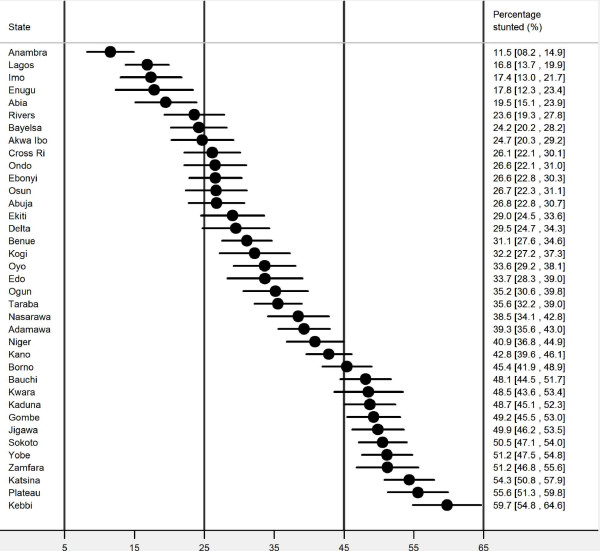
Caterpillar’ plot of childhood stunting in Nigeria, 2008.

Figure 1 shows the estimated and ranking of childhood stunting for each of the 37 states, together with the associated 95% confidence intervals (CIs). The percentage of children with stunting ranged from 11.5% in Anambra state to as high as 60% in Kebbi State. The same Figure 1 is showing the ranking of states with respect to childhood stunting. Anambra and Lagos states had the least numbers; 11.5% and 16.8% respectively while Yobe, Zamfara, Katsina, Plateau and Kebbi had the highest (with more than 50% of their under-fives having stunted growth)

### Funnel plot

**Figure 2:**
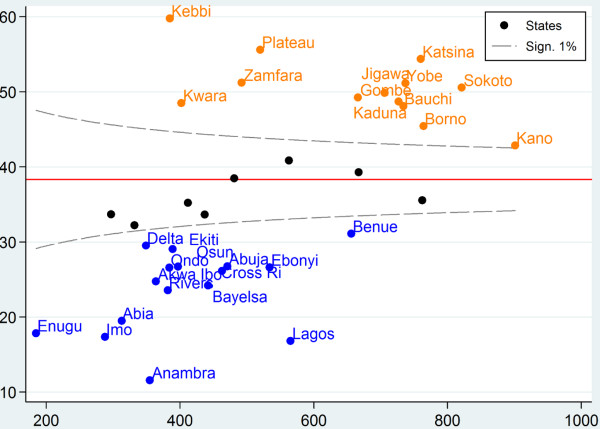
Funnel plot of childhood stunting in Nigeria, 2008.

As shown in Figure 2, there is a wide variation in the percentage of stunting between the 37 states. The funnel plot identifies only eight (22%) states within the 99% control limits (demonstrating common-cause variation). Thirteen states (35%) were above the upper control limit (higher than the national average) and sixteen (43%) states were below the lower control limit (lower than the national average), indicating special cause variation.

### Spatial autocorrelation

**Figure 3:**
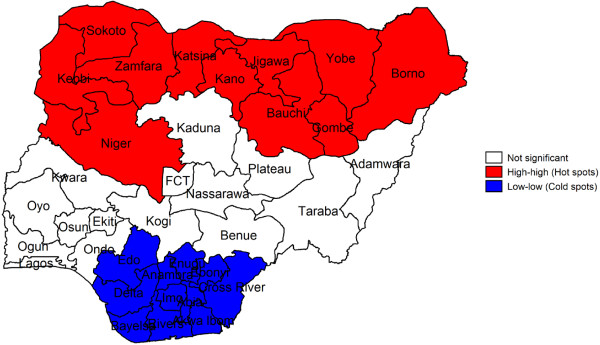
LISA cluster map for childhood stunting, 2008.

Figure 3 shows the results of Local Moran I for childhood stunting. The results show statistically significant spatial autocorrelation between states (Moran's I = 0.775, p < 0.001), such that states in red colour belong to the hot spot clusters and the states in blue belong to cold spot clusters.

## Discussion

We examined the variation in childhood stunting in Nigeria by using within-data analysis triangulation method and found that there is a wide variation in the childhood stunting in Nigeria, with twenty-eight states (78%) out of the thirty-seven states in the country showing evidence of special-cause variation which merits further investigation to identify possible causes. However, about eight states representing 22% are consistent with common-cause variation which is a variation that is consistent with and within the national average (stable process). We found that childhood stunting is highest in the northern part of Nigeria.

Looking at the league table, it appears that childhood stunting in Nigeria is highest in Kebbi, Plateau, Katsina, Zamfara, Yobe, Sokoto, Jigawa, Gombe, Kaduna, Kwara, Bauchi, Borno and Kano states. These states were also the outliers identified through the funnel plots. This implies from the control chart that the variations identified in these states were due to a ‘special cause’.

The ESDA identified Kebbi, Niger, Katsina, Zamfara, Yobe, Sokoto, Jigawa, Gombe, Bauchi, Borno and Kano states as the ‘hot spots’. These are states that share similar neighbourhood or area characteristics and have high childhood stunting correlating with each other. The findings of this study support those of other similar studies which indicated that living in certain geographical areas has a negative effect on health outcomes [1-3,20,21]. Results from ESDA were not significant for Kaduna, Plateau and Kwara states. Kebbi, Niger, Katsina, Zamfara, Yobe, Sokoto, Jigawa, Gombe, Bauchi, Borno and Kano states were consistently identified from all methods used. From this study, it seems that childhood stunting is a key issue in the northern states and this should call further investigation to understand the special cause associated with these states when compared with others.

However, Anambra, Lagos, Imo, Enugu, Abia, Rivers, Bayelsa and Akwa Ibom states appear to have the lowest childhood stunting in Nigeria—all three analytical methods identified these states. The inference that could be drawn from this study is that there are ‘special practices’ from these states that could be identified as good practice. This should also warrant further studies to identify what practices or process that are particular to these states. However, one likely special practice that may be responsible for the low level of childhood stunting in some states in the southern part of Nigeria could be the preschool/school feeding programmes of some state governments in which proteinous foods are given freely to pupils. Deeply rooted in poverty and deprivation, stunting is a nutritional problem that affects mainly developing countries like Nigeria with varied levels of poverty rate and health deprivation index across different geopolitical zones. Northern states have higher poverty rates and health deprivation index than their southern counterparts which may explain for the observed variation seen in this study. Future studies should examine the observed special-cause variation using the generic pyramid model [22]. The generic pyramid model is adapted from industry and comprised checking the data first, then the case-mix, the resources and the work process, and finally check the individual health care practitioners [22]. Thus, the vast majority of problems (95%) may be due to work system and not from individual healthcare practitioners [22].

A number of methods have been used for assessing the performance and exploring the variation in healthcare outcomes in the past [23-28]. These methods, individually, have their shortcomings and consequently lead to concerns on reliability and acceptance of results from each of these methods. Most literatures have mentioned insufficient risk adjustments as a problem with some methods [7,26,28]. Marshall and Mohammed [29] explored the use of control charts in monitoring mortality outcomes across hospitals in the United Kingdom. The authors summed up their findings that case-mix adjustments may not be essential for longitudinal monitoring.

The study can be criticized for using an indirect measure (synthetic life table) for measuring childhood stunting. However, due to the fact that in low- and middle-income countries, it is hard to obtain reliable data from birth registers; synthetic life table generated from cross-sectional data could be considered a good proxy for childhood stunting data. Teller et al. [30] were able to verify the validity and reliability of the DHS for monitoring childhood stunting in developing countries. These analyses give a snapshot of childhood stunting in Nigeria using the 2008 DHS; in the course of further investigation of risk factors, there is the need to report on trends with (statistical significance) as well.

Despite these limitations, the study strengths are significant. It is a large, population-based study with national coverage. In addition, data of the DHS are widely perceived to be of high quality, as they were based on sound sampling methodology with high response rates of about 98%. To the best of our knowledge, this is the first study to use ‘within data triangulation’ to explore childhood stunting as a health outcome in sub-Saharan Africa. The within-data triangulation was developed using three different analytical methods, namely league table, funnel plots and spatial cluster analysis. This has potential to enhance the validation and reliability of our analysis through cross verification of methods used.

## Conclusions

Childhood stunting is high in Nigeria and varied significantly across the different states. The northern states have a higher proportion than the southern states. There is an urgent need for studies that will explore factors that may be responsible for these special cause variations in childhood stunting in Nigeria.

## Competing interests

The author(s) declare that they have no competing interests.

## Authors’ contributions

VTA and OAU conceived the study. VTA and OAU completed all statistical analyses. VTA, OAU and OMM drafted the manuscript. VTA, OAU and OMM contributed to the discussion. VTA and OAU revised the manuscript. All authors have read and approved the final manuscript.

## Pre-publication history

The pre-publication history for this paper can be accessed here:

http://www.biomedcentral.com/1471-2458/13/361/prepub
